# The Effect of Species *Soybean Vein Necrosis Orthotospovirus* (SVNV) on Life Table Parameters of Its Vector, Soybean Thrips (*Neohydatothrips variabilis* Thysanoptera: Thripidae)

**DOI:** 10.3390/insects13070632

**Published:** 2022-07-14

**Authors:** Asifa Hameed, Cristina Rosa, Edwin G. Rajotte

**Affiliations:** 1 Department of Entomology, Pennsylvania State University, University Park, State College, PA 16802, USA; uvu@psu.edu; 2 Plant Pathology and Environmental Microbiology, Pennsylvania State University, University Park, State College, PA 16802, USA; czr2@psu.edu

**Keywords:** soybean thrips, life table, soybean vein necrosis virus, life expectancy, thrips biology

## Abstract

**Simple Summary:**

Soybean vein necrosis, caused by soybean vein necrosis virus (SVNV) is an important viral disease of soybeans that can be seed borne or insect vectored. This plant viral disease affects seed qualitative parameters, including seed oil content. Increased damage is observed in late planted soybeans. The disease is widespread, and almost all soybean-growing states in USA are affected. Globally, SVNV is reported in Canada, the United States, Egypt and Pakistan. In order to manage the disease, it is important to understand the vector’s biology and the effect of SVNV on life table parameters (survival, longevity, mortality, doubling time, generation, rate of intrinsic increase) of vector soybean thrips, which can help to establish pest management predictive models. We used an age-stage two-sex life table estimation model to define the effect of SVNV on the life parameters of male and female soybean thrips. Overall, we found that SVNV infection increased viruliferous thrips survival, longevity, gross reproduction rate, life expectancy and decreased population doubling time. Overall viruliferous thrips benefit from SVNV infection and transmission due to better survival, longevity and increased fitness.

**Abstract:**

Soybean vein necrosis orthotospovirus (SVNV: Tospoviridae: Orthotospovirus), the causal agent of soybean vein necrosis disease, is vectored by soybean thrips *Neohydatothrips variabilis* (Beach, 1896), and to a lesser extent by five other thrips species. There is increasing incidence of soybean vein necrosis (SVN) disease in all soybean growing states in the United States, Canada, Egypt and Pakistan, necessitating a study of the system’s ecology and management. We addressed the effect of SVNV on the life table parameters of the vector. We used an ‘age-stage two-sex’ life table approach, which provided detailed life stage durations of each larval instar and adults (both sexes). Our results showed that the intrinsic rate of increase (r), finite rate of increase (λ) and mortality index (qx) were higher in the infected population, while the net reproduction rate (Ro), cumulative probability of survival (lx) and gross reproduction rate (GRR) were lower in the uninfected population. Overall, in both infected and uninfected populations, the number of eggs producing haploid males via arrhenotoky ranged from 9–12 per female. Male to female ratio was female biased in the infected population. Overall, our study provided evidence that virus infection, by decreasing the population doubling time, could enhance the virus and vector populations in soybeans.

## 1. Introduction

Soybean vein necrosis orthotospovirus (SVNV, Tospoviridae: Orthotospovirus) is thecausal agent of a seed [1] and thrips-borne [2] disease of soybean present in North America [2,3,4,5,6], the Middle East [7,8] and Pakistan [9], which may cause significant reduction in oil content [5,10]. Although only 2% of plant viruses are transmitted by thrips, thrips transmitted *Orthotospoviruses* can cause severe economic losses to ornamental, fruit and vegetable crops around the world [11,12,13]. SVNV was first identified in Tennessee in 2008; later, in 2013, the disease was reported in all soybean growing states of the United States, and in 2015, it was reported in Canada. The dispersion of disease was quick. The effect of SVNV on soybean yield and quality are not well-known except for a few studies that document 0.1% reduction in oil content and fatty-acids profiling [1,5,10]. An unwanted amino-acid’s (linolenic acid) concentration is increased due to SVNV, while no significant effects on oleic acid were observed [5]. In Indiana, on an SVNV-infected double crop, the oil content was significantly reduced, while the protein content was increased. Understanding the tripartite relationships among the host, viruses and thrips will allow the development of better prediction of disease and pest pressure as well as more efficient pest management strategies [14,15].

Soybean thrips *Neohydatothrips variabilis* (Beach, 1896) is an important vector of SVNV virus [2,16,17]. Thrips acquire SVNV during initial larval stages (L1–L2) through feeding on SVNV-infected plants [17]. The acquired SVNV in thrips is gradually passed to the salivary glands from where it is transmitted to other host plants [17]. Soybean vein necrosis virus is a persistent, circulative and propagative virus [17]. In addition to *N. variabilis* (Beach, 1896), SVNV is transmitted by five other species of thrips (Thysanoptera: Thripidae): *Frankliniella occidentalis* (Pergande, 1895), *F. tritici* (Fitch, 1855), *Caliothrips phaseoli* (Hood, 1912), *F. fusca* (Hinds, 1902) and *Megalurothrips sjostedti* (Trybom, 1908) [2,7,16]. Among all thrips vectors found on soybeans, *N. variabilis* can transmit SVNV at a rate 2.5 times higher than *F. fusca* and 10 times higher than *F. tritici* [16]. Seasonality varies among vector species in Pennsylvania USA. At the onset of the growing season, eastern flower thrips (*F. tritici*) are present in higher numbers, but their population decreases later in the season [18]. In northern areas of North America, the most efficient vector, *N. variabilis*, arrives later and gradually becomes a prominent thrips species on soybean in the late season [18]. This may be a reason for increased incidence of SVNV in the later season.

Virus infections may impact insect vector development [19,20,21] and survival [15,22], which can influence virus transmission [23,24]. Orthotospoviruses can change vector biology and life history traits. In the life history, we can determine each stage duration oflarvae, pre pupae, pupae and adult (both sexes), including egg incubation time [25].

Another orthotospovirus (species *Tomato spotted wilt orthotospovirus*, TSWV) has differential effects on the males and females of *F. occidentalis*. TSWV infection enhances the production of vitellogenin protein, which is required for yolk development and enhances the survival of resulting immature nymphs [26]. *F. occidentalis* reared on TSWV-infected plants had improved survival, but took significantly longer to develop compared to uninfected thrips. It is still not clear why the virus and vector interactions are variable depending on the virus-vector pairing, but knowledge of the effect of particular viruses on their thrips vectors needs to be determined to be useful in disease management.

A life table analysis can provide critical information about changes in vector life history parameters caused by virus infection. In another study, the western flower thrips infected with impatiens necrotic orthotospovirus (INSV) [27] thrips reared on infectedplants had reduced survival, reproductive potential and increased development time. The information gained through life table studies can help predict vector population dynamics, which is necessary for the development of integrated pest management (IPM) strategies [25].

In this study, we explored the effect of SVNV on the life table of *N. variabilis* by comparing life tables of SVNV-infected and uninfected male and female thrips. Life table elements included stage-specific mortality percentage, survival index, rate of intrinsic increase, gross reproductive rate (GRR), life expectancy (Ex), population doubling time(DT), intrinsic birth rate (B), intrinsic death rate (B-rm), generation time (T) and finite rate of increase in number (λ). Keough et al., (2016) described the effect of SVNV on life history traits of *N. variabilis* viz., total larval stage duration L1-P2, number of eggs laid 7 days after post-eclosion (This included the mating time, pre-oviposition period and post ovipositionperiod) and hatching time, but different aspects of life history viz., duration of each larval and pupal stage, adult total fertility until the time of death and total adult duration until the time of death were not studied [16]. We provided a more detailed life table including information about duration of 1st instar larvae (L1), 2nd instar larvae (L2), pre-pupae (P1), pupae (P2) and adult life including fertility.

## 2. Materials and Methods

### 2.1. Collection of Insects and Infected Thrips Population Development

To develop an SVNV-infected thrips population, about twenty *N. variabilis* were collected from soybean fields at the Penn State Russell E. Larson Agricultural Research Station at Rock Springs, Pennsylvania Furnace, Pennsylvania, in August, 2016. Soybean thrips are predominant on the upper canopy leaves during the day. Hence, the upper canopy leaves were thoroughly checked. Thrips were collected using a camel’s hair brush and placed in a plastic jar covered with muslin cloth. Fresh soybean leaves were also placed in these jars. Adult thrips were immediately transferred to the laboratory and released on 10 plants, which were placed in insect rearing cages (L24.5 × W24.5 × H24.5 cm). These insect rearing cages were placed in an environmental growthchamber at a temperature of 25 + 2 °C and humidity of 80% L:D 14:10 (Conviron, Winnipeg, MB, Canada). Plants started showing characteristic SVNV disease symptoms, one month after thrips were released. In order to confirm virus presence in thrips and plant populations, real-time PCR was performed.

### 2.2. Development of Uninfected Thrips Population

For the purpose of development of uninfected thrips population, thrips were collected from virus-free plants. An uninfected thrips population was developed by collecting pupae from a field-derived population and releasing them on healthy soybean plants individually on each plant covered with the top half of a clear 1.5 L bottle preventing thrips from moving to other plants according to a method developed by Keough et al. [16]. After one month, plants/insects were confirmed to be uninfected using real time-PCR. The uninfected insects were pooled to initiate a colony. Before the life table studies, the colony was again checked for virus presence. This was precautionary because the virus is transmitted through the seed as well as via vector [1].

### 2.3. Confirmation of Virus Infection through Real Time PCR in Plants

Virus infection status of the plants (infected and uninfected) was determined by extracting RNA from symptomatic leaves using Direct-zol RNA Microprep Kit (Zymo Research, Irvine, CA, USA). For virus status in thrips, RNA was extracted from groups of twenty-five thrips using an RNeasy Mini Kit (Qiagen, Hilden, Germany). RNA quality was checked on a Nanodrop 2000 (Thermo Fisher Scientific, Waltham, MA, USA). From the RNA, cDNA was synthesized using the High-Capacity cDNA Reverse Transcriptase kit (Applied Biosystems, Foster City, CA, USA) with >0.31–2.0 µg RNA as input. To test virus presence in the infected and uninfected thrips population, a real time PCR assay was performed following the procedure of the PowerUpTM SYBR TM kit (Applied Biosystem, Foster City, CA, USA) using 2 µL of cDNA as template. As an internal control for soybean, the soybean eukaryotic elongation factor 1-beta GmELF1-b (NCBI accession number; XM_003545405) and associated primers were used [16]. To test for SVNV presence, previously designed primers [16] to amplify parts of the virus genomic segments coding for the nucleocapsid and non-structural silencing suppressor were used (Table S1). Real time PCR conditions were 95 °C (for 2 min), followed by 40 cycles of 95 °C (10 s), 55 °C (30 s) and final melting curve (65 °C to 95 °C) at 0.5 °C increase each 5 s.

### 2.4. Confirmation of Virus Infection through Real Time PCR in Thrips

Virus presence or absence in uninfected thrips was tested using 25 uninfected thrips (one poll two technical replicates). The same primers (Table S1) were used to detect SVNV, and the cytochrome p450 gene (accession number: EF523586) as *N. variabilis* internal control, and the results are described in Table S2.

### 2.5. Life Table Studies

For the life table studies, twenty pairs of adult thrips from each colony were released onto an individual uninfected hydroponic trifoliate leaf kept in separate small rearingcups and placed in a growth chamber at 25 + 2 °C, photoperiod 16:8 (L:D). The adults were given one week for oviposition and then removed. After hatching, one hundred F1 first instar larvae were transferred into individual petri-dishes containing a fresh soybean leaf. Insect growth was recorded at 24-h intervals to detect molting. The duration ofeach instar was recorded at the molt, and sex was determined upon adult eclosion. Again, for the F2 generation, male and female pairs were placed on hydroponic leaves for matingand egg laying. The number of neonate thrips was counted daily to determine fertility and fecundity over the period until parental death. We determined the survival and mortality in adult thrips using *Thrips palmi* (Karny, 1925) survivorship as a guide. We chose10, 15 and 20 days to calculate lx [28], so we assumed it may be at least 10 days in *N. variabilis*. All the terminologies used in the paper and their definition are provided in Table S3.

### 2.6. Statistical Analysis

The thrips life history was analyzed using an age stage-specific two-sex Lt horizontal life table analysis [29,30]. Life table survival plots were constructed using Kaplan-Meir statistical analysis in SPSS software (IBM Corp, Armonk, NY, USA, Version 23.0) [31]. The differences among treatments (infected vs. uninfected) were assessed using life table plots and log-rank (Mantel-Cox) Chi-squared (*c*2) tests. We compared all factor levels in a single test by pooling over strata to test the equality of survival curves. An event was defined as 1 = dead and 0 = alive. The infected population was coded infected = 1 and uninfected = 2. Survival plots were created for each population and each thrips growth stage. Mean individual reproductive potential per day was the total number of nymphs produced per day (fx). Survivorship of each instar was compared using one-way analysis of variance (SPSS, IBM Corp., Armonk, NY, USA, Version 23.0). Fertility of females was compared using Levene’s *t* test (SPSS, IBM Corp Armonk, NY, USA, Version 23.0). For adults, a two-way ANOVA determined the duration of the adult instar for each sex. The duration of each immature stage (larvae stage 1 (L1), larvae stage 2 (L2), pre-pupae (P1) and pupae (P2)) and adult was determined using Kaplan-Meir life table calculations (SPSS, IBM Corp USA, Version 23.0). Mean, standard error, lower-upper limits, *c*2-value, F value and *p*-value were determined. A *c*2 test was used to measure the difference between the population means. Graphs were developed through Kaplan-Meir and ggplot2 (R version 3.5.3).

## 3. Results

### 3.1. SVNV Infection Status in the Infected Strain and Confirmation of Uninfected Population

The real-time PCR on the NP and NSs genomic segments showed that the SVNV was present in the infected thrips population and plants, while it was not observed in uninfected plants and insects (Table S2).

### 3.2. Impact of Virus on Thrips Biological Growth Parameters Such as Development Time, Adult Longevity and Fertility

**Figure 1:**
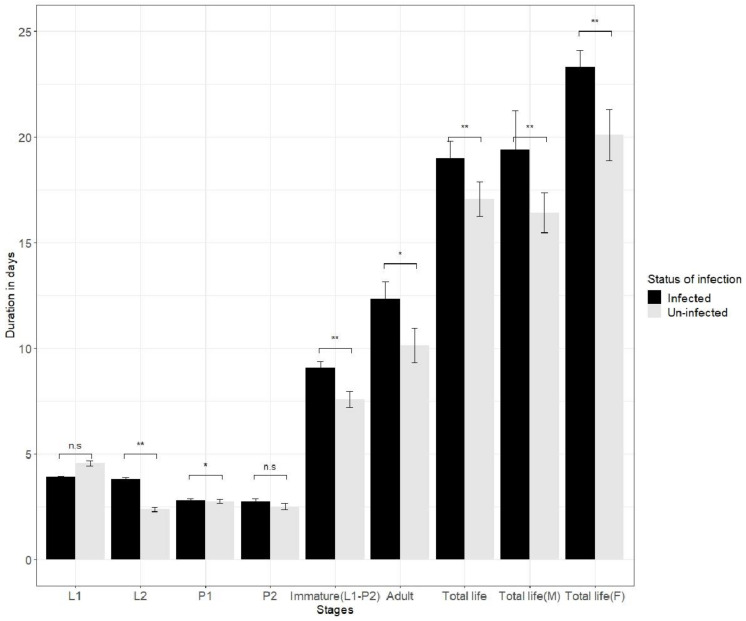
Effect of soybean vein necrosis orthotospovirus (SVNV) on development time, adult longevity and total lifespan of uninfected and infected *Neohydatothrips variabilis*. Here L1, L2, P1 andP2 denotes larval stage 1, larval stage 2, pre-pupae, and pupal stage, respectively. The comparison between infected and uninfected populations of the same stage used the log rank (Mantle-Cox) chi-squared test in SPSS at 5% level of significance. The symbols *, ** and n.s. represents significant (*p* < 0.05), highly significant (*p* < 0.01) and non-significant (*p* > 0.05) difference in infected and uninfected populations comparisons.

Comparisons between infected and uninfected thrips’ development time, longevity and fertility showed differences for some life stages. Infection by SVNV did not affect first instar (L1) duration (uninfected L1 = 4.547 + 0.118 days, infected L1 = 3.902 + 0.047 days, *c*2 = 1.24; *p* = 0.264; Total N = 200; N (censored) = 179; df = 1) (Figure 1). Here, total N is total number of organisms that entered in particular stage, while N (censored) is total number of organism that completed the stage and molted to the next instar. The second instar (L2) duration was significantly lower in the uninfected population (uninfected L2 duration = 2.376 + 0.131 days, infected L2 duration = 3.80 + 0.082 days, *c*2 = 13.38, *p* = 0.00, Total 199 N = 179; N(censored) = 155; df = 1) (Figure 1).

The pre-pupal stage (P1) duration was significantly lower in the uninfected population {uninfected P1 = 2.746 + 0.100; infected P1 = 2.795 + 0.081 days, (*c*2 = 3.916, *p* = 0.048 and N (total) = 155, N(censored)= 143) (Figure 1). The duration of the P2 pupal stages was notdifferent (uninfected P2 = 2.753 + 0.119 days; infected P2 = 2.508 + 0.142 days *c*2 = 0.075; *p* = 0.785; N(total) = 143; N(censored) = 118; df = 1).

Total immature life (TIL) and adult life (AL) duration was lower in the uninfected population (TIL duration uninfected = 7.589 + 0.374 days; TIL duration infected = 9.088 + 0.297 days, *c*2 = 7.245, *p* = 0.007, N(total) = 200; N(censored) = 118; AL duration uninfected = 10.137 + 0.816 days, AL duration infected = 12.343 + 0.822 days, *c*2 = 4.045 *p* = 0.007; N(total) = 118; N(censored) = 0; df = 1), respectively (Figure 1).

Total adult life duration in adult females was significantly lower in the uninfected 218 population (17.059 + 0.808 days) compared to the infected population (19.00 days + 0.812, 219 *c*2 = 4.858; *p* = 0.028; total N = 118; N(censored) = 0; df = 1). The total adult male longevity was lower in uninfected male compared infected male {(uninfected adult male duration = 7.589 + 0.374, infected adult male duration = 9.088 + 0.297), *c*2 = 7.245; *p* = 0.007; total N = 118; N(censored) = 0); df = 1} (Figure 1). Overall, the infected thrips survived longer.

**Figure 2:**
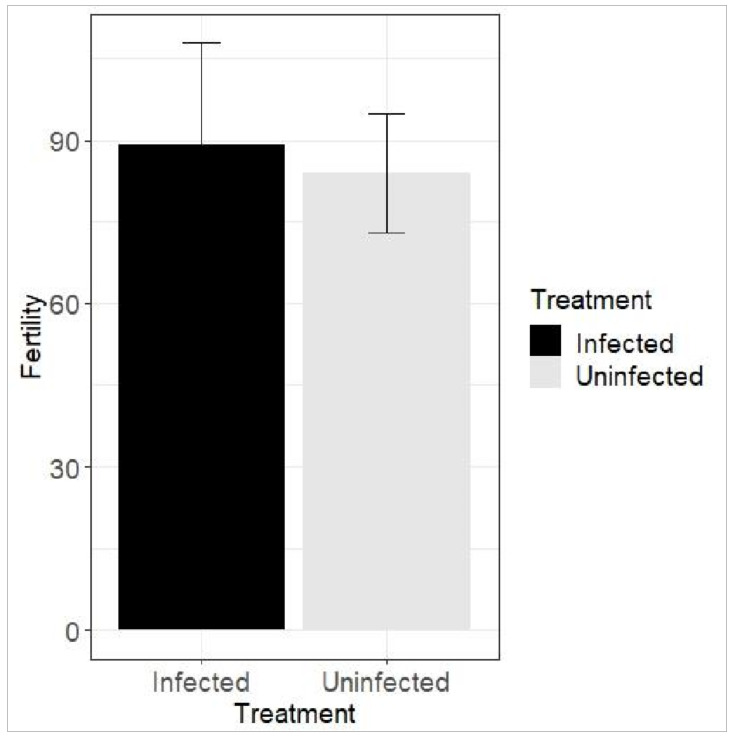
Effect of SVNV on the fertility of *Neohydatothrips variabilis* (Beach.). Mean number of eggs between two populations were compared through independent sample *t* test (*p* = 0.026, *t* = 0.284, 230 SEM = 18.54, 10.912; df = 45).

Levene’s *t* test revealed that fertility (average number of eggs laid by a female over a life time) was not significant (infected and uninfected population, F = 5.337; *p* = 0.026, T = 0.284) in the infected thrips (89.21 + 18.541 eggs; N = 21; df = 45) compared to uninfected thrips (83.96 + 10.912 eggs; N = 26; df = 45) (Figure 2).

### 3.3. Impact of SVNV on the Life Table Parameters

#### 3.3.1. Survival and Mortality Parameters

The probability of survival and mortality in the infected and uninfected *N. variabilis* populations was different depending on viral infection (Table 1). Infected thrips survived longer, and mortality was lower as compared to uninfected thrips. The experiment started with 100 thrips in each treatment. The probability of death (qx) in the L1 was greater in the uninfected population compared to infected population (uninfected = 0.15; infected = 0.06). Similarly, the probability of death was greater in uninfected L2 compared to infected L2 (uninfected = 0.21; infected = 0.06). The probability of death in uninfected P1 was significantly higher than in the infected population (probability of death in uninfected = 0.09, probability of death in infected = 0.068). Similarly, mortality in pupae in the uninfected population was higher than the infected population. Overall, the mortality was higher in the uninfected *N. variabilis* population compared to the infected population (Table 1). The cumulative probability of survival at the beginning of time interval (lx) was higher in the infected population. So, the probability of survival to the age x (npx) was higher in the infected population viz., the probability of survival of infected L1, L2, and P1 in the infected population was 0.94, 0.936, 0.932, respectively, compared to the uninfected L1, L2, P1 and P2, which was 0.85, 0.788, and 0.910, respectively (Table 1). The probability of survival in the 249 pupal stage was higher in the uninfected population compared to the infected population (Table 1). The value of npx for adult males and adult females *N. variabilis* in the uninfected population was lower compared to the infected population (Table 1).

**Figure 3:**
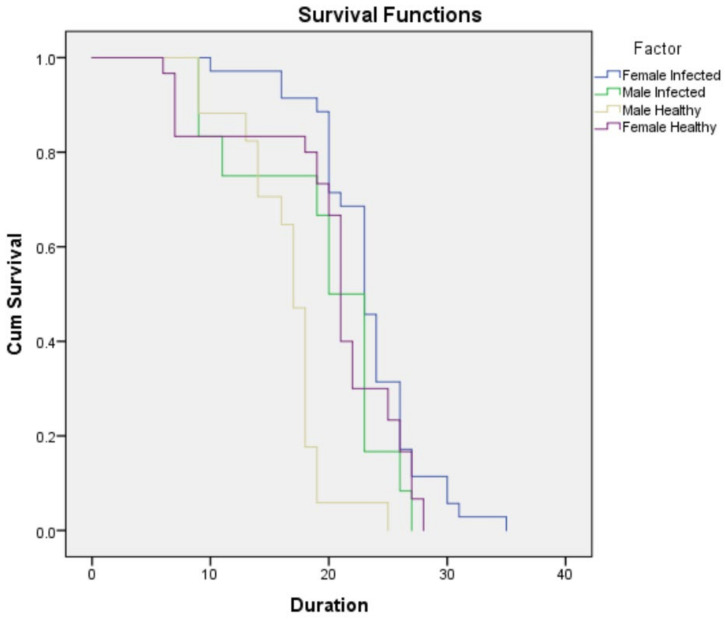
Kaplan-Meir survival graph showing the survival of the SVNV-infected and uninfected *Neohydatothrips variabilis* populations.

The percent probability of death was higher in the uninfected population in all instars except the fourth instar (Table 1). The average proportion alive at that age (Lx) was higher in the infected strain compared to uninfected thrips (Table 1). Overall, the average proportion alive in the uninfected population was lower than in the infected population (Table 1 and Figure 3).

The cumulative number of days lived beyond the minimum age of 10 days (Tx) was lower in the uninfected versus infected populations (Table 1).

#### 3.3.2. Effect of SVNV on Thrips Reproduction

**Figure 4:**
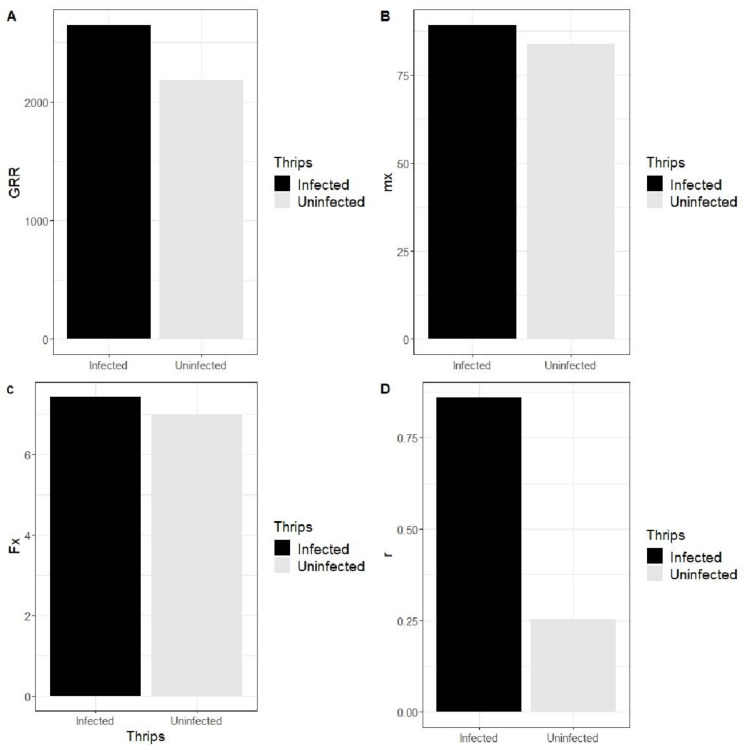
Effect of SVNV on reproductive parameters of *Neohydatothrips variabilis*. (**A**) Effect of virus on Gross reproductive rate (GRR). (**B**) Effect of virus on the average eggs produced per female (mx). (**C**) Effect of virus on the average number of eggs laid per day by reproductive female (fx). (**D**) 284 Effect of virus on the rate of intrinsic increase.

Reproductive parameters, including gross reproduction rate (GRR), average number of offspring produced (mx), life expectancy (Ex), average fertility (mx) and intrinsic rate of increase (r) varied between uninfected and infected populations. Gross reproduction rate (GRR) is the sum of all offspring produced by a reproductive female and was higher in infected populations compared to uninfected populations (Figure 4). The average number of offspring produced (mx) by reproductive females and the female life expectancy was lower in the uninfected population compared to the infected population (Figure 4). The average fertility (mx) was 83.96 eggs in the uninfected population and 89.21 eggs in the infected population. Eggs produced per day (Fx) were 6.997 in the uninfected population and 7.434 in the infected population. The rate of intrinsic increase (r) was 0.859 in the infected population and 0.253 in the uninfected population (Figure 4). The rate of intrinsic increase was higher in the infected population because a higher number of females survived. The presence of virus increased the survival of the females.

#### 3.3.3. Effect of SVNV on Population Growth Parameters

**Figure 5:**
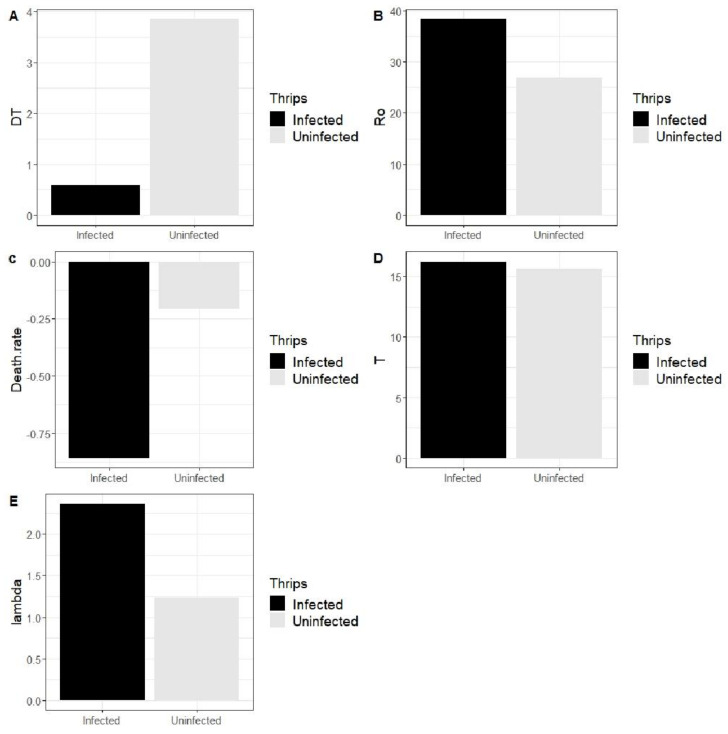
Effect of SVNV on population growth parameters of *Neohydatothrips variabilis*. Effect ofSVNV on (**A**) population doubling time (DT). (**B**) Ro. (**C**) The death rate (b-rm). (**D**) Generation time (T). (**E**) Finite rate of increase in number (lambda λ).

Population doubling time (DT) is defined as the time a population takes to double in number. It is dependent upon fertility, sex ratio and longevity of the immature instars. Population DT was lower in the infected population compared with the uninfected population (Figure 5). In the uninfected population, the DT was 3.851 days while in the infected population it was 0.595 days. The intrinsic birth rate (B) was 0.001 in the uninfected population compared to 0.002 in the infected population. Intrinsic death rate(b-rm) was −0.209 in the uninfected population and −0.857 in the infected population. However, precise generation time (T) was 15.642 days in the uninfected population compared to 16.25 days in the infected population. Finite rate of increase in number (λ) was 1.288 in the uninfected population and 2.361 in the infected population (Figure 5). Ro was 26.87 in the uninfected population and 38.36 in the infected thrips population (Figure 5). Overall, due to virus infection, population doubling time (DT) and death rate decreased, while the finite rate of increase in number and generation time increased.

## 4. Discussion

Overall, we found that SVNV increased the duration of the immature stages and the adult stage in infected thrips compared to the uninfected population. Infected thrips had significantly longer L2 and P1 stages, but did not have a significantly longer L1 or P2. This may be due to the fact that pupal stage is non-feeding. In *F. occidentalis*, TSWV infection significantly increased the duration of L2 [27,32]; however, duration of P2 was not affected. Because *F. occidentalis* do not feed during the pupal stage due to molting, the virus may not have an effect on pupal duration [33]. One explanation of increased survival of infected thrips may be an increase of amino acids in the virus infected plants which results in increased access to amino acids and consequent insect survival [34].

We also found that total immature development time/duration for *N. variabilis* was longer in the SVNV-infected population. In another study, Sether (1991) found that total immature duration was also lower in uninfected *F. occidentalis*, compared to infected thrips exposed to TSWV [32]. The results of our work are similar to other studies that showed that infection of western flower thrips and soybean thrips by TSWV and SVNV, respectively, increased insect performance, longevity and survival [16,35]. We hypothesize that since SVNV infection increases longevity and fitness of thrips, SVNV-infected thrips may be able to avoid the predators like TSWV-infected thrips can avoid predatory mites [22]. TSWV positively affected the survival and development of *F. occidentalis* larvae raised on mechanically inoculated plants [15]. However, these results contrast with Chen et al., (2014), who reported that *Thrips palmi* development time did not differ significantly in watermelon silver mottle virus-infected and healthy larvae. *T. palmi* took significantly longer to grow on uninfected plants compared to the infected plants. The contrasting results suggest that effects of orthotospovirus vary among virus vector combinations resulting in variable effects on insect development time and fecundity.

Adult life and total life were significantly higher in the SVNV-infected population (Figure 2). However, virus infection decreases the survival and reproductive potential of the TSWV infected *F. occidentalis* [32,35]. We found that SVNV infection increased adult life duration and reproductive potential of *N. variabilis*. We also observed that lx, the cumulative probability of survival, was higher in the infected population while the probability of death was lower in the infected population (Table 1). In western flower thrips, TSWV infection can increase survival [35]; however, other studies have found that soybean thripssurvival does not increase with SVNV infection [16]. The results of the present work suggest that SVNV infection increases the fitness of *N. variabilis*, which is similar to results obtained with other viruses and other thrips species [20,33,36,37,38,39,40] and in contradiction to other studies that reported the presence of virus in other thrips species decreased the fitness or did not affect it at all [20,27,41,42,43,44]. Alterations in vector behavior (longer feeding and oviposition) and fitness (survival and duration of each insect stage) associated with virus-induced physiological changes (nutritional chemistry) in host plants in many cases may seem to favor pathogen dispersal [45,46,47].

In the present study, we found that gross reproduction rate (GRR) was higher in the SVNV-infected population (Figure 5). The expectancy of life (ex) was lower in male as compared to females, but infection increased life expectancy in both males and females. We expected males to have a shorter life expectancy because thrips are haplodiploid, and females usually live longer than males and continue to lay eggs [48,49]. Ro is the replacement rate. Overall, if Ro < 1, the population is shrinking. If Ro > 1, the population is increasing. If Ro = 1, it means that population is constant. In the present study, the value of Ro (age specific maternity) was lower in the uninfected population (26.87 compared to 38.36 in the infected population). Ro is a product of lx and mx values since the probability of survival and the number of eggs produced were lower in the uninfected population, hence the value of Ro was also lower in the uninfected population.

Since the work was done under lab conditions, we could not assess external mortality factors (predator or parasitoid effect on population abundance) other than natural mortality. Eggs produced (mx) (fertility) was 83.96 in the uninfected females and 89.21 in the SVNV-infected ones. The direction of virus effects [50] on fecundity was similar to Keough et al. [16]. It is still not clear whether these differences are due to the direct effect of the virus on the thrips or to the indirect effects of feeding on virus-infected plants.

Overall, infected *N. variabilis* survive longer, produce higher numbers of offspring, have reduced doubling time and suffer from reduced mortality due to SVNV infection. Work on proteomics and transcriptomics of infected and uninfected thrips populations should be carried out to understand the processes involved in increasing fitness of viruliferous populations. Integrated pest management practices, including the selection of resistant cultivars and utilization of thrips natural predators to control the phytophagous thrips species, should be developed.

Comparative life tables of infected and uninfected *N. variabilis* populations showed that SVNV-infected thrips survive longer, produce a higher number of offspring and have reduced mortality and higher survival proportion as compared to the uninfected thrips. Rate of intrinsic increase was greater in the infected population compared to the uninfected population. The uninfected thrips population took longer to double as compared to the infected population. Overall, soybean thrips benefit from infection of SVNV and population doubling time was reduced in the infected population.

## 5. Conclusions

Comparative life tables of infected and uninfected *N. variabilis* populations showed that SVNV-infected thrips survive longer, produce a higher number of offspring and have less mortality and higher survival compared to uninfected thrips. Infected females survived longer compared to uninfected females and males. SVNV-infected females produced a nominally higher number of offspring compared to uninfected females. Gross reproduction rate and rate of intrinsic increase was higher in the infected population compared to the uninfected population. The uninfected thrips population took longer to double as compared to the infected population. Overall, soybean thrips benefit from infection of SVNV through better survival, reduced death rate and decreased population doubling time.

## Figures and Tables

**Table 1:** Life table comparison of uninfected and SVNV-infected *Neohydatothrips variabilis* populations in larval stages and adults.

Status of Infection	Stage	Proportion of Individual Surviving (Lx)	The Probability of Surviving the Period (Npx)	lx*px	Percent Probability of Death 100 qx	Cumulative Number of Days Lived beyond Age × Tx
	L1	1	0.85	0.85	15	19.26
	L2	0.85	0.788	0.67	21.176	15.4
	P1	0.67	0.91	0.61	8.955	12.08
	P2	0.61	0.836	0.51	16.393	8.93
Infected Thrips	Adult male age = (10 days)	0.19	0.105	0.02	89.474	1.05
	Adult male age = (15 days)	0	0	0	100	0
	Adult female age = (10 days)	0.627451	0.719	0.451	28.125	8.93
	Adult female (age = 15 days)	0.23	0.348	0.08	65.217	1.74
	Adult female (age = 20 days)	0.08	0	0	100	0
	L1	1	0.94	0.94	6	24.19
	L2	0.94	0.936	0.88	6.383	20.52
	P1	0.88	0.932	0.82	6.818	16.97
Uninfected Thrips	P2	0.82	0.817	0.67	18.293	14.36
	Adult male age = (10 days)	0.22	0.409	0.09	59.091	4.09
	Adult male age = (15 days)	0.09	0	0	100	0
	Adult female age = (10 days)	0.45	0.956	0.43	4.444	12.11
	Adult female (age = 15 days)	0.43	0.512	0.22	48.837	2.56
	Adult female (age = 20 days)	0.22	0	0	100	0

## Data Availability

Data is contained within the research article and supplementary files.

## Supplementary Materials

The following supporting information can be downloaded at: https://www.mdpi.com/article/10.3390/insects13070632/s1, Table S1: List of the primers used for detection of virus; Table S2: Relative quantification of SVNV in the infected and uninfected plants and thrips population; Table S3: List of acronyms and terminologies used in the paper.
