# Supplementary material for: The Effect of Species Soybean Vein Necrosis Orthotospovirus (SVNV) on Life Table Parameters of Its Vector, Soybean Thrips (Neohydatothrips variabilis Thysanoptera: Thripidae)
Source: Insects. 2022 Jul 14;13(7):632. doi: 10.3390/insects13070632 (PMC9324745; doi:10.3390/insects13070632)
Supplement: Supplementary file 1 [file insects-13-00632-s001.zip › insects-1760878-supplementary.pdf]

Table S1. List of the primers used for detection of virus.

Gene	Gene title	Primer sequence (5` to 3`, forward/reverse)	Genbank acc.no	Product size
GmELF1-b	Glycine max Elongation factor 1	ACTCTGCACTCACCAC TGCC/AGGAAAGCTTG GAGCAAGTTGAG	XM_003545405	247
SVNV-NP	Nucleocapsid Protein	GGAAGCTTACCCCTTC TGGC/ACTCCTCTCATT TGGGGTGC	GU722322	239
SVNV-NSs	Non-structural silencing protein	TGATGACCACGAAAG GGTCT/AACAACCAGA TGCGGAACCT	GU722319.1	191
NV COI	N. variabilis Cytochrome C oxidase subunit	GGATTTATTGTTTGAG CACACCAC/TCCTGTC AATCCTCCTAATGTGA	EF523586	216

Table S2. Relative quantification of SVNV in the infected and uninfected plants and thrips population.

Treatment/ biological replicate	Gene	Avg. Cq	Melting temperature	Virus detected/not
Healthy thrips	SVNV-NSs	NaN	None	N.D
Healthy thrips	SVNV-NSs	NaN	None	N.D
Infected Thrips	SVNV-NSs	23	79	D
Infected Thrips	SVNV-NSs	19	79.5	D
Water	SVNV-NSs	NaN	None	N.D
Healthy thrips	SVNV-NP	NaN	None	N.D
Infected Thrips	SVNV-NP	22	80.5	D
Infected Thrips	SVNV-NP	19.93	80.5	D
Water	SVNV-NP	NaN	None	N.D
Healthy thrips	NV COI	21	75.5	D
Healthy thrips	NV COI	21	75.5	D
Infected Thrips	NV COI	25	75.5	D
Infected Thrips	NV COI	21	75.5	D
Water	NV COI	NaN	None	N.D
Healthy plant	SVNV-NSs	NaN	None	N.D
Healthy plant	SVNV-NSs	NaN	None	N.D
Healthy plant	SVNV-NSs	NaN	None	N.D
Infected Plant	SVNV-NSsSVNV-NSs	14	79	D
Infected Plant	SVNV-NSs	13	79.5	D
Infected Plant	SVNV-NSs	12	79.5	D
Healthy plant	SVNV-NP	NaN	None	N.D
Healthy plant	SVNV-NP	NaN	None	N.D
Healthy plant	SVNV-NP	NaN	None	N.D
Infected Plant	SVNV-NP	12	80.5	D
Infected Plant	SVNV-NP	12	80.5	D
Infected Plant	SVNV-NP	13	80.5	D
Healthy plant	GmELF1-b	26	81.5	Host plant gene
Healthy plant	GmELF1-b	25	81.5	Host plant gene
Healthy plant	GmELF1-b	33	81.5	Host plant gene
Healthy plant	GmELF1-b	26	81.5	Host plant gene
Healthy plant	GmELF1-b	26	81.5	Host plant gene

Healthy plant	GmELF1-b	29	81	Host plant gene
Healthy plant	GmELF1-b	26	81.5	Host plant gene
Infected Plant	GmELF1-b	22	81.5	Host plant gene
Healthy thrips	SVNV-NSs	NaN	None	N.D
Healthy thrips	SVNV-NSs	NaN	None	N.D
Infected Thrips	SVNV-NSs	23	79	D
Infected Thrips	SVNV-NSs	19	79.5	D

Table S3. List of acronyms and terminologies used in the paper.

Acronym	Formula	Description
Ndx		The number of individuals from the original cohort die during the interval (x to x+n), out of the total number of individuals at the beginning of the table.
Lx		Proportion of individual surviving; the cumulative probability of survival at the beginning of time interval.
Npx	npx=1-nqx	The probability of surviving the period.
lx+1	lx*px	
100 qx		Percent probability of death
Lx	Lx≈(lx + l x+1)/2	Average proportion alive at a particular age
Tx	Lx+Lx+1+….+Lx+n	
	cumulative number of days lived beyond age x	
GRR		
	Σmx	Gross reproduction rate. It is sum of all eggs produced during the time period by reproductive females
‘ex’		
	(Tx/lx)	The average number of days yet to be lived by members surviving to a particular age (x)
Ro	Σlx.mx	Ro is the replacement rate. If Ro < 1, the population is shrinking. If Ro >1 the population is increasing. If Ro=1 it means that population is constant.
‘dfx’		Mortality factor responsible for dx
mx		Eggs produced per surviving stage or average eggs produced per female
Fx		Eggs produced per day or per each age
R	InRo/Tc	Intrinsic rate of natural increase of population. rate of intrinsic increase = Σe7-rmx. Lxmx. When the r=0, the population is stable. If r>0 the population is increasing, if r<0 the population is decreasing or shrinking.
DT	(ln 2)/rm	Population doubling time
B	1/Σe-rmxlx	Intrinsic birth rate
‘dr’	b-rm	Intrinsic death rate
T	Σx*(lx.mx)/Σ(lx*mx)	Generation time

Lambda(λ)	Antilog erm	Finite rate of increase in number
Ndx		The number of individuals from the original cohort die during the interval (x to x+n), out of the total number of individuals at the beginning of the table.
Npx	npx=1-nqx	The probability of surviving the period.
